# Papular acne keloidalis nuchae treatment success using follicular unit excision

**DOI:** 10.1016/j.jdcr.2023.07.013

**Published:** 2023-07-25

**Authors:** Sanusi Umar, Raveena Khanna

**Affiliations:** a Department of Medicine, Dermatology Division, University of California, Los Angeles, California; b Division of Dermatology, Harbor-UCLA Medical Center, Torrance, California; c Dr U Hair and Skin Clinic, Manhattan Beach, California; d Department of Dermatology, Howard University College of Medicine, Washington, District of Columbia

**Keywords:** acne kelidalis nuchae, African, African American, African hair, Afro-textured, all-purpose punch, alopecia, coily hair, curly hair, follicular units, follicular unit excision, follicular unit extraction, fue, hair follicles, hair graft, hair loss, hair restoration surgery, hair transplant, transection rates

## Introduction

Acne keloidalis nuchae (AKN) is a chronic debilitating and suppurating cicatricial alopecia that affects men of color but is rarely reported in European-descended Whites and women. Factors influencing treatment choice and outcomes reflect a wide variation in morphologic forms, including dispersed or merging papules/nodules, atrophic or raised plaques, and tumorous masses. Wide-ranging scalp disease distribution patterns, including a varying degree of nape zone affectation to more extensive scalp distribution and the presence or absence of other cicatricial scalp diseases, including folliculitis decalvans (FD), are also contributing factors.^1^, ^2^, ^3^, ^4^ Various studies have shown that laser hair removal can achieve long-term remission in papular AKN.^2^^,^^5^ However, there are mitigating conditions that restrict the applicability or effectiveness of laser hair removal (LHR) in AKN treatment. As previously reported by the lead author (SU),^2^ effective LHR in AKN requires that the vertical cross-sectional height of the lesion does not exceed 3 mm above the surrounding skin. Although the average length of scalp hair follicles ranges from 2 to 4 mm, with the coarsest ones averaging around 5 mm, the hair reduction laser with the furthest reach—the neodymium-doped yttrium aluminum garnet laser, denoted Nd:YAG - has a penetration depth of 5-7 mm.^2^ Furthermore, lasers are also ineffective in removing grey hair,^6^ making them unsuccessful in AKN lesions containing these hairs. In addition, some patients are not receptive to collateral hair damage or loss and expected patchy alopecia that occurs when targeting AKN lesions by LHR, especially when targeting widely dispersed AKN lesions.

Follicular unit excision (FUE) is a method of extracting single hair follicular unit groups using small punches that avoid the creation of large wounds and scars, thus preserving the patient’s ability to shave their heads with minimal footprints.^7^ For this reason, FUE is a favored method of hair transplantation.^7^^,^^8^ We report 3 patients with treatment success using FUE to target the hair follicles at the center of visible AKN papules while avoiding collateral damage in the form of hair loss to surrounding hair or visible scarring.

## Material and methods

Patients were seen at a Los Angeles-based dermatology clinic specializing in scalp and hair disorders. The patients had histologically proven (Supplemental Fig 1, available via Mendeley at https://doi.org/10.17632/vykj97vdgg.1) papular AKN lesions that persisted despite treatments with medications. All patients rejected LHR due to the possibility of patchy hair loss and preferred targeted hair elimination using FUE.

After obtaining written informed consent, the affected zone was prepped and numbed using local anesthetics: lidocaine 1% with 1:100,000 epinephrine. FUE was performed to target the removal of single follicular units in the center of discernible AKN papules while taking care to preserve hair follicles not invested in AKN lesions.

**Fig 1:**
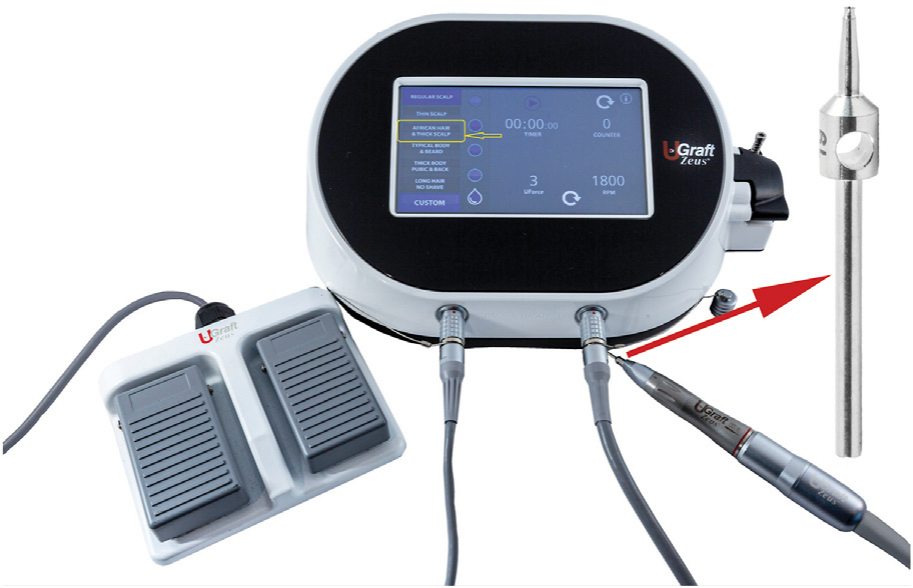
The UGraft Zeus device showing a handpiece, foot pedal, and a console with a dashboard highlighting (*yellow*) the thick-firm scalp preset selection and the torque (UForce) and rotational speed (RPM) dials. Inset shows a closer view of the punch (*Red arrow*). *RPM*, Revolution per minute.

FUE was performed using an all-purpose FUE device consisting of a punch - The Intelligent Punch and its driver, Dr UGraft Zeus (Dr U Devices Inc) (Fig 1). The punch and punch driver incorporate several proprietary design features, including punch texturing, flaring, configurations, and manipulation of movement forms and torque forces tunable to changing skin thickness and firmness. The latter features allow navigation of the subdermal curved courses of hair with minimal assistance from the surgeon.^8^ Depending on the average diameter of the AKN papules, punch sizes varied between 19G, 18G, and 17G.

**Fig 2:**
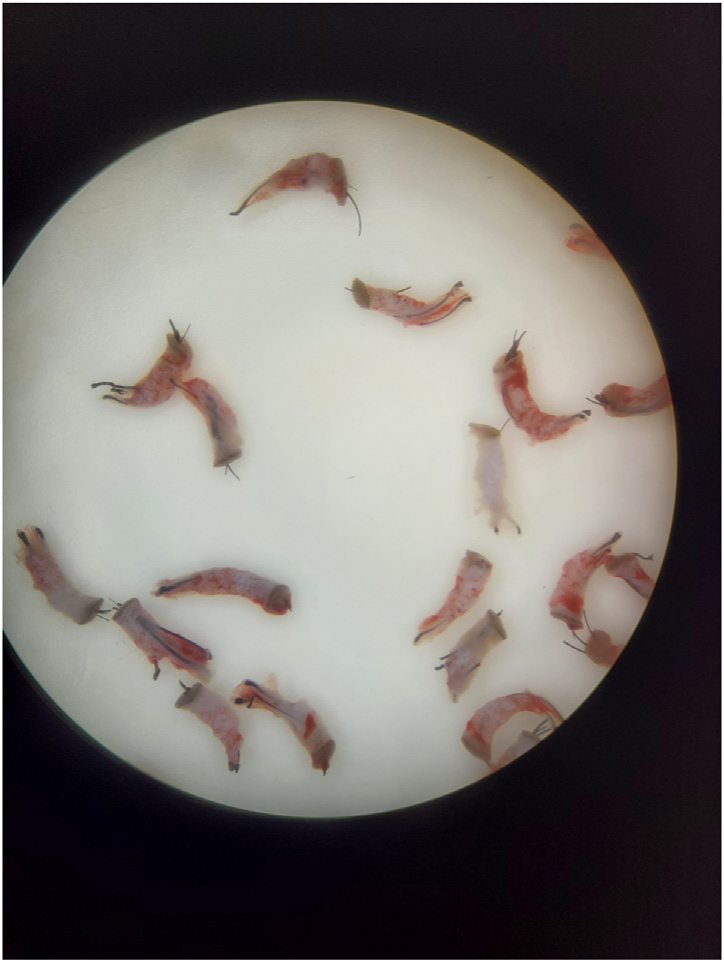
Curly follicular unit grafts excised from papular AKN lesions of an African patient using a specialized FUE punch device. *AKN*, Acne keloidalis nuchae; *FUE*, follicular unit excision.

The dashboard setting for hard thick skin was chosen with torque and movement settings favorable to the hard and thick skin conditions inherent to AKN lesions (Fig 1). As shown in the Supplemental Video, available via Mendeley at https://doi.org/10.17632/vykj97vdgg.1 demonstrating the method of use, the surgeon engulfs the hair follicle at the center of the AKN lesion at its exit point, holding the punch between 60 and 90 degrees to the skin surface. Next, the surgeon allows the punch to penetrate the skin under the weight of the handpiece or with minimal force. Upon complete separation from surrounding anchoring tissues, the follicle is observed to partially pop out of the skin. The follicles/grafts (Fig 2 and Supplemental Fig 2, available via Mendeley at https://doi.org/10.17632/vykj97vdgg.1) are pulled out using a jeweler’s forceps, occasionally assisted by minimal dissection of residual tissue tethers using 23-gauge hypodermic needles armed on a 3-cc syringe.

**Fig 3:**
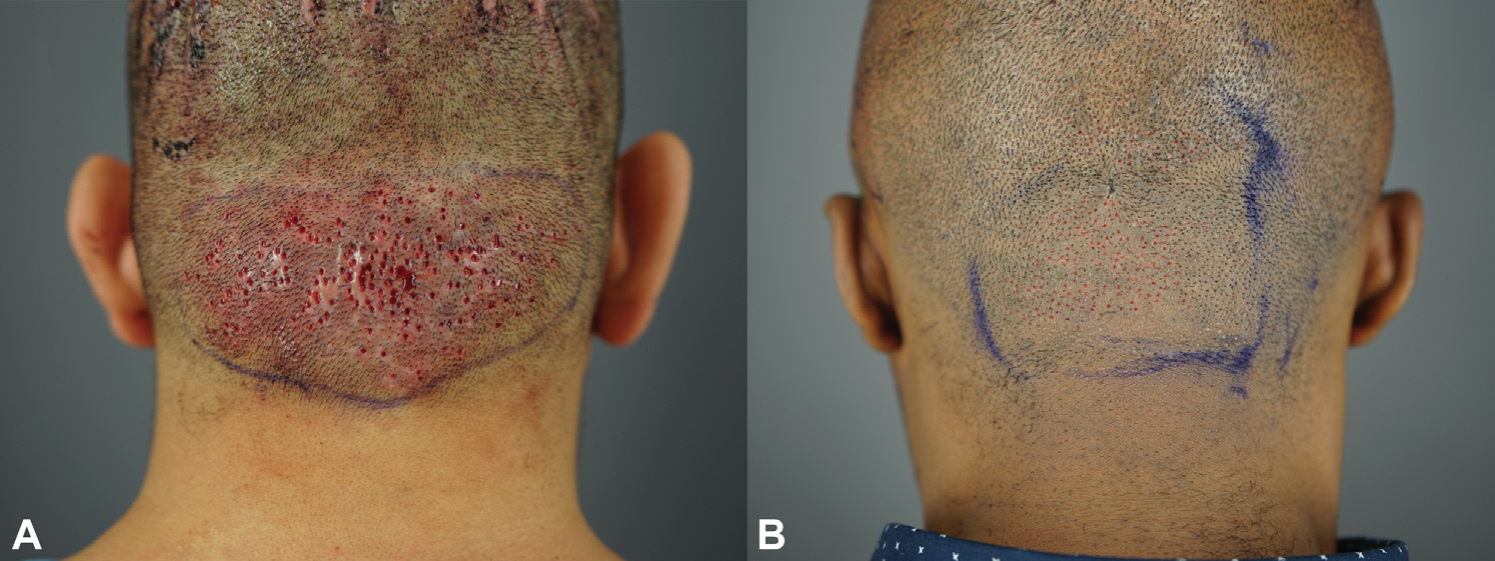
Hispanic male patient seen with tiny wounds secondary to FUE procedure, taken immediately after surgery for papular AKN (**A**). African male patient with tiny wounds secondary to FUE procedure, taken immediately after surgery for papular AKN (**B**). *AKN*, Acne keloidalis nuchae; *FUE*, follicular unit excision.

Since the FUE method is fast, up to 2500 grafts can be removed in a single session from a wide area of the scalp; a speed of 400 extractions per hour is typical. The extraction zone is left open to heal by second intention healing since the small extraction wound sizes are not expected to create untoward scarring. Given that the FUE wounds are minuscule (Fig 3, *A* and *B*), they heal by second intention with cosmetically insignificant footprints, even after shaving the area. Either Aquaphor or bacitracin ointment is applied to the extraction zone twice daily.

## Patient 1

**Fig 4:**
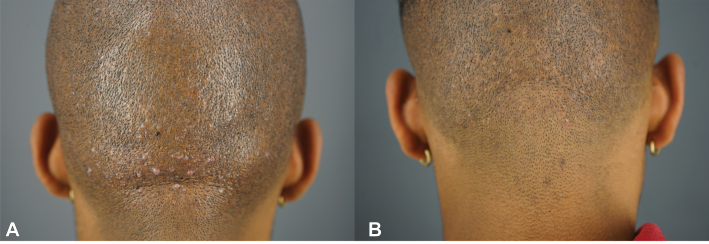
26-years-old African American male with AKN Class 2 papules negative for folliculitis decalvans pre-FUE procedure (**A**). At 4 m post-FUE procedure, his nape area remained clear (**B**). *AKN*, Acne keloidalis nuchae; *FUE*, follicular unit excision.

A 26-years-old African American man sought treatment for a 5-years history of AKN papules predominantly affecting the nape area (AKN class 2 merging papules positive for folliculitis decalvans)^1^ and associated pruritus (Fig 4, *A*). Prior treatments included topical steroids, antibiotics, and topical exfoliants to control disease progression, but the condition persisted. Upon further examination, the patient also had perifollicular infundibulo-ishmic lymphocytoplasmic infiltrates with fibrosis (PIILIF) on trichoscopy and histology, affecting his entire normal-appearing scalp (NAS) zones.

He was commenced on minocycline 100 mg twice daily before the procedure and continued this regimen for 6 weeks postoperatively. His AKN lesions were treated using the described FUE methods, and 90 grafts were extracted from the affected region. His nape area remained clear 9 months after the procedure (Fig 4, *B*). His PIILIF-affected scalp zones were treated using topical tacrolimus 0.1% ointment daily and a topical botanical – Gashee (FineTouch Laboratories Inc).^9^

## Patient 2

**Fig 5:**
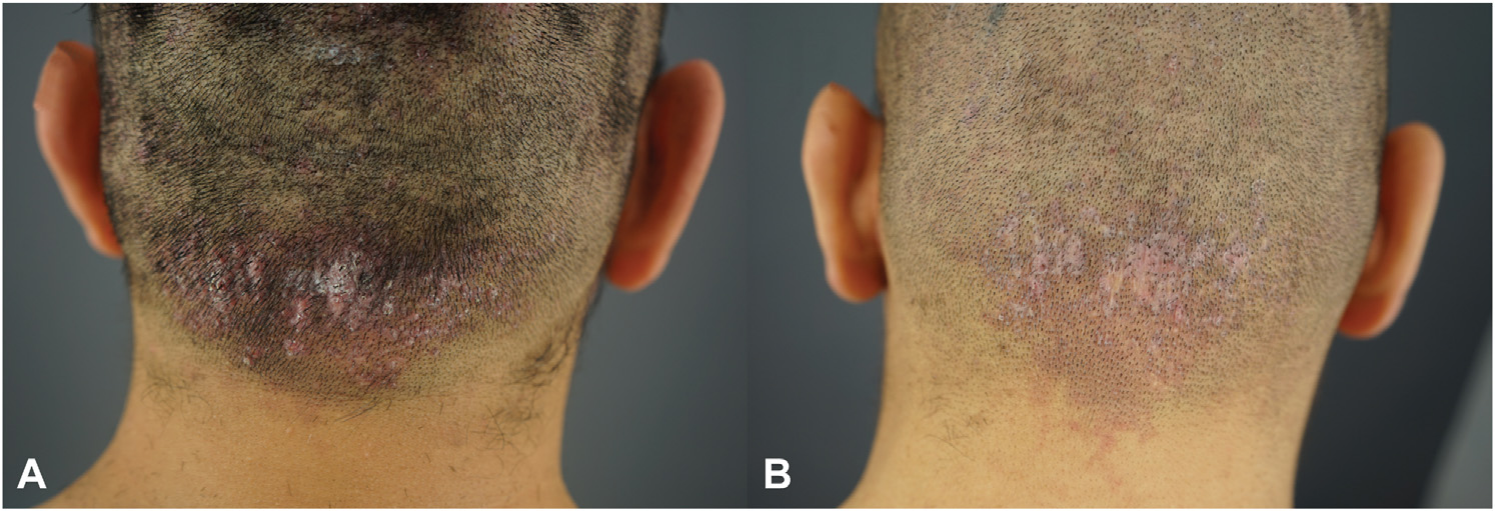
37-years-old Hispanic male—with AKN Class 2 merging papules positive for folliculitis decalvans—as seen before FUE procedure at nape area (**A**). At 4 m post-FUE procedure (**B**), the area remained clear even after discontinuing all previously prescribed medications. *AKN*, Acne keloidalis nuchae; *FUE*, follicular unit excision.

A 37-years-old Hispanic man sought treatment for both a folliculitis decalvans (FD) plaque occupying his entire crown and a greater than 10-years history of merging AKN follicular papules (AKN class 2 merging papules positive for FD)^1^ (Fig 5, *A*). He was also found to have PIILIF involving the rest of his scalp. Previous treatments targeting his FD included topical steroids, intralesional steroid injections, and isotretinoin, but the conditions (FD and AKN) persisted. He had some FD symptomatic relief during a 2-years course of Humira (adalimumab), but this was eventually discontinued upon improvement, ultimately resulting in a recurrence of FD disease. His AKN lesions were not affected by the listed treatments.

The AKN lesions were treated using the described FUE methods, and 135 grafts were extracted. Four months after the procedure, the area remained clear of lesions (Fig 5, *B*) with no disease recurrence at 9 months postoperatively, despite discontinuing previously prescribed medications.

The FD was treated separately by surgical excision with second-intention healing aided by guarded high-tension sutures. The PIILIF-affected NAS zones were treated using topical tacrolimus 0.1% ointment daily and the topical botanical mentioned above.^9^

## Patient 3

A 42-years-old African man with Norwood 5 male pattern baldness initially presented to the clinic seeking consultation for hair transplantation surgery but was found to have a greater than 10-years history of follicular-papular AKN. His disease (AKN Class 2 papules negative for folliculitis decalvans)^1^ was mostly confined to his nape area. Previous treatment with topical steroids and antibiotics somewhat controlled disease progression, but the lesions continued to wax and wane for years.

A 19G all-purpose device punch was used to retrieve 3477 grafts from his beard and the back and sides of his scalp. His AKN lesions were treated using the described FUE methods, and 120 grafts were extracted from the affected nape area. Healing occurred without sequelae and showed lesional clearance. At 18-months follow-up, the nape remained clear of AKN lesions, despite discontinuing his previous preoperative topical treatment regimen.

## Discussion points

We report AKN treatment success with long-term remission of more than 6-12 months using targeted FUE of the hair follicles at the center of AKN papules. Patient selection criteria included hair-bearing AKN papules (follicular papules), laser hair removal that was either rejected or unsuitable, and failure or unsuitability of conservative drug therapy. Long-term remission was achieved for all patients in 1 treatment session.

A previous report described large skin biopsy punches (3 mm and greater) in excising AKN lesions and tufted hairs in a series of 6 treatments.^10^ However, large punches would cause large circular scars, which would not be suitable for mild AKN cases where the threshold for acceptable scarring is low compared to severe AKN plaques. Furthermore, targeting scattered, tufted hairs in AKN are not likely to result in long-term remission because the few tufted follicles in the AKN plaque do not account for most of the diseased zone, where only a few hairs are visible candidates for punching out. We believe that efficacy and long-term remission would require eliminating all intralesional hair, which is surgically possible only in lesions with visible hairs projecting from them. Thus, AKN plaques and masses with vast alopecic zones may not be as suitable for treatment by FUE since the culprit hairs responsible for most of the active lesions are embedded within the AKN tissue and are not practically accessible for removal by FUE or the use of large punches. Because all 3 patients met the selection criteria and had papules with hairs projecting from them, targeting those follicles for removal was consistently effective, and the results were long-term.

The choice of the FUE technique used is also significant for minimizing transection. This is especially important since transected follicles would result in ingrown hair and folliculitis, which can have the unwanted effect of flaring AKN rather than improving it.^11^ Hair invested within AKN lesions especially presents challenges to FUE for several reasons. First, most AKN patients are of African descent, thus possessing curlier hair scalps, posing a challenge to conventional FUE devices.^1^^,^^12^ Second, in patients having noncurly hair, the straight hair course is subject to distortion by the scarring process of the encompassing AKN lesion. Lastly, FUE is generally more challenging in skin types that are thicker and firmer, which is inherent to AKN, where the follicles are invested in thickened, fibrous/scarred tissue. As described in a previous publication,^8^ the all-purpose device optimized for subdermal hair curliness, skin thickness, and firmness was beneficial in minimizing graft transection. Given the ability to perform this FUE procedure quickly, on any part of the scalp, with minimal untoward cosmetic consequences, this process could be adopted to resolve scalp-wide involvement of AKN papules in qualified candidates.

Besides their classic AKN lesions, patients with AKN have scalp-wide involvement of PIILIF, which may represent a subclinical disease.^13^ This should be treated separately, however as should the frequently associated FD.^1^

The limitations of this report include the retrospective nature and small sample size. A larger cohort of patients would be optimal to confirm and extend our findings.

## Conclusion

In 3 patients where AKN lesions invest svisible hair follicles, FUE hair removal was an effective therapy that resulted in long-term disease remission with esthetically satisfactory results.

## Conflict of interest

Dr Umar has ownership shares in Dr U Devices Inc and FineTouch Laboratories Inc, patents issued to Dr U Devices Inc (patent no: USPTO US887684B2 & USPTO US9095368B3), and patent applications (patent no: PCT WO2019203882A1). The remaining authors have no conflicts of interest in this work to declare.
